# Agreement between CDC/NHSN surveillance definitions and ECDC criteria in diagnosis of healthcare-associated infections in Serbian trauma patients

**DOI:** 10.1371/journal.pone.0204893

**Published:** 2018-10-04

**Authors:** Olivera Djuric, Ljiljana Markovic-Denic, Bojan Jovanovic, Vesna Bumbasirevic

**Affiliations:** 1 Faculty of Medicine, University of Belgrade, Belgrade, Serbia; 2 Institute of Epidemiology, Faculty of Medicine, University of Belgrade, Belgrade, Serbia; 3 Centre for Anaesthesiology, Clinical Centre of Serbia, Belgrade, Serbia; University of Maryland School of Medicine, UNITED STATES

## Abstract

After three national point prevalence studies (PPS) of healthcare associated infections (HAI) conducted in Serbian acute care hospitals using US (CDC/NHSN) surveillance definitions, Serbia is about to switch to European (ECDC) criteria for the purpose of the fourth HAI PPS. The aim of this study was to compare the US and the European HAI definitions in Serbian trauma intensive care unit (ICU). Prospective surveillance was performed at two surgical-trauma ICUs of the Emergency department of Clinical Center of Serbia. HAIs were prospectively diagnosed by experienced clinician and epidemiologists using both types of HAI definitions simultaneously. The level of agreement between two case definitions was assessed by Cohen’s kappa statistic (k). Of 406 patients, 107 (26.3%) acquired at least one HAI (total of 107 according to US definitions and 141 according to European criteria). For microbiologically confirmed pneumonia agreement was k = 0.99 (95% CI, 0.96–1.00) and for clinically defined k = 0.86 (95% CI, 0.58–1.00). Agreement for bloodstream infections (BSI) was 0.79 (CI 95%, 0.70–0.89). When secondary BSI was excluded from the European classification, (30.9% of all BSI), concordance was k = 1.00 and when microbiologically confirmed catheter related BSI were reported separately as recommended by latest ECDC protocol update, (20.0% of all BSI), concordance was 0.60 (CI 95%, 0.41–0.80). No agreement was found between CLABSI and CRI while slight agreement was found when compared CLABSI and CRI3 (k = 0.11; 95%CI, 0.0–0.22). Agreement for overall UTI was moderate (k = 0.66; 95%CI, 0.53–0.79) while for microbiologically-confirmed symptomatic UTI was perfect (k = 1.00). For CAUTI good agreement was observed (k = 0.77; 95%CI, 0.34–1.0). Microbiological confirmation of PN and UTI should be stimulated and comparison of BSI should be done with emphasis on whether secondary BSI is included.

## Introduction

Surveillance of health care associated infections (HAI) is a valuable tool in decreasing infection rates in health-care settings and improving the patient safety [1,2,3]. In order for surveillance to be efficient and to result in valid inter-country and inter-network infection rate comparison, standardized definitions and methodology are necessary [4].

First uniform definitions of nosocomial infections for the surveillance purposes were developed in 1970s by the Centers for Disease Control and Prevention (CDC)[5] and starting from 1988 were officially recommended to hospitals participating in CDC National Nosocomial infection Surveillance Sistem (NNIS)[6]. Since then, CDC definitions were modified for surgical site infections (SSI) in 1992 [7], ventilator-associated pneumonia (VAP) in 2004 [8], bloodstream infections (BSI) and clinical sepsis in 2008 [9], and urinary tract infections (UTI) in 2010 [10]. In 2013 CDC’s National Healthcare Safety Network (NHSN) launched new criteria for identifying HAI cases, while in 2015 set of changes for specific localizations were published [11,12].

In Europe, during the 90ies, national surveillance networks were, due to different objectives of their infection control practices, adopting and modifying CDC definitions or developing their own criteria, which resulted in unstandardized methodology and limited comparability of results. Harmonization of the national HAI surveillance systems and consequently harmonization of the HAI definitions across Europe was initiated by Hospitals in Europe Link for Infection Control through Surveillance (HELICS) project and continued by European Centre for Disease Prevention and Control (ECDC) through the Healthcare-Associated Infections Surveillance Network (HAI-Net)[13, 14]. The protocol for first EU-wide point prevalence survey (PPSs) was published in 2012 by ECDC, comprising standardized surveillance methodology and HAI definitions [15]. In October 2016 updated ECDC protocol was published for the purpose of the second EU-wide PPS which this time included several EU enlargement countries of European region that are not member states yet, among which Serbia [16].

In Serbia, CDC/NHSN surveillance definitions were translated in 1998, updated in 2008, and implemented for the purposes of both, point prevalence studies and prospective surveillance studies [17]. So far, three national PPS studies of HAIs in acute care hospitals were conducted in Serbia (in 1999, 2005 and 2010) using these definitions and their updated versions. Fourth national PPS is about to be undertaken and this time as a part of the second EU-wide PPS. For this purpose ECDC definitions have been translated and introduced to infection control professionals [18].

Following the transition from American to European definitions, the aim of this study was to assess the agreement between American (CDC/NHSN) surveillance definition and European (ECDC) criteria for diagnosis of the most common HAIs in ICU. To assess this we used population of ICU trauma patients as a pilot population since ICU patients are at highest risk of acquiring HAI, and moreover, those who sustained trauma are considered not to have infection on the admission.

## Materials and methods

### Study design and patients

A prospective patient-based, single-center surveillance study was conducted at two 24-beds trauma-surgical ICUs of the Emergency Department of Clinical center of Serbia (CCS) in a year and a half period (from November 2014 to April 2016). Emergency department of CCS is a tertiary teaching hospital and the sole adult tertiary care trauma center serving the southern and central areas of Serbia. All consecutive adult trauma patients admitted to one of two trauma-surgical ICUs during the study period and who spent more than 48h in ICU were eligible for the study. On admission data regarding the patients’ socio-demographic characteristics, comorbidities, severity of trauma (Injury Severity Score, ISS) and severity of disease [Acute Physiology and Chronic Health Evaluation II score (APACHE II) and Sequential Organ Failure Assessment score (SOFA)] were recorded.

The study was approved by the Ethics Committee of the Clinical Centre of Serbia (No 1358/19) and by the Ethics Committee of the Faculty of Medicine at the University of Belgrade, Serbia (No 29/X-5). Written informed consent was obtained from all of the patients enrolled or the patient’s next to kin. Trauma patients’ capacity to provide informed consent was assessed by attending intensive care specialists (BJ, VB) by using clinical assessment of patient’s status. If patient had reduced or no capacity to understand and give informed consent to participate in the study, next to kin were required to give informed consent on behalf of the patients. All procedures performed in studies involving human participants were in accordance with the ethical standards of the institutional and/or national research committee and with the 1964 Helsinki declaration and its later amendments or comparable ethical standards.

### Surveillance

Active surveillance for HAI was performed prospectively by an experienced physician (BJ) and infection control epidemiologists (LMD and OD). Patients were prospectively assessed for the presence of infection by daily visits and examining the records of patients, clinical course, radiology, laboratory and culture results. Only first episodes of specific HAI localizations were considered for the analysis. CDC/NHSN and ECDC definitions were simultaneously applied to diagnose presence of HAI, assess HAI localization and its device association (for PN, BSI and UTI) according to both definitions [19, 20].

### Statistical analysis

Categorical data was presented as number with percentages. The level of agreement between criteria when diagnosing HAIs was assessed by pairwise comparisons using Cohen’s kappa statistics (k) which show how much better the agreement is than that of chance, with a value of zero indicating no agreement better than chance and value of one indicating perfect agreement [21]. Ninety-five percent confidence interval (95%CI) for kappa was calculated using the formula k ± 1.96xSE. Statistical analysis was performed using SPSS version 17.0 software (IBM-SPSS Inc, Armonk, NY, USA).

## Results

### Description of trauma patients

Cohort of 406 trauma patients (312 males and 94 females) were followed for 5258 ICU days. Median length of stay was 9 days (range, 2–131). Table 1 shows patients demographic, clinical and trauma characteristics. Median (IQR) age was 49 (35) years. Less than half of the patients had any comorbidity (n (%) = 165 (40.6%)). Of these, 94 (57%) patients had underlying cardiac disease, 12 (7.3%) patients had chronic pulmonary disease, while 6 (3.6%) patients were immunocompromised. Considering severity of trauma and patients’ state at the admission, they were mostly severely injured according to mean ISS score of 19.9 ± 8.5 and had moderately severe medical condition at admission (mean APACHE II score 10.6 ± 6.9).

**Table 1:** Characteristics of patients.

**General characteristics**		
Median (IQR) age		49 (35)
Male gender		312 (76.8)
Comorbidities	Cardiac disease	94 (57.0)
	Chronic pulmonary disease	12 (7.3)
	Diabetes mellitus	16 (9.7)
	Chronical infection	6 (3.6)
	Immunocompromised	6 (3.6)
	Carcinoma	10 (6.1)
	Other	21 (12.7)
	**Total**	165 (40.6)
Number of comorbidities	1	111 (27.3)
	2	32 (7.9)
	≥ 3	22 (5.4)
**Clinical characteristics**		
Type of admission	Emergency department	244 (60.1)
	Operating room	79 (19.5)
	Other hospital	76 (18.7)
	Medical ward	7 (1.7)
Mean Glasgow Coma Score		12.2 ± 3.9
	≤ 8	87 (21.5)
	8–13	56 (13.8)
	≥ 13	262 (64.7)
Intubation	At scene	12 (3.0)
	Emergency department/Operating room	160 (67.8)
	Intensive care unit	14 (3.4)
	Other hospital	50 (12.3)
	**Total**	236 (58.1)
Mean APACHE II score		10.6 ± 6.9
Median (IQR) SOFA score		4 (3)
Mean Injury Severity Score		19.9 ± 8.5

Values are n (%) unless otherwise stated.

IQR, Interquartile range

### Definitions comparison

Prior to calculation of the agreement level between US and European definitions, both HAI criteria were reviewed and compared by researchers (Table 2). Case definition for ICU-acquired HAI differs depending on the presence of the indwelling device. Although both definitions agree that symptoms need to occur earliest on the day 3 for infection to be considered as hospital-acquired, according to ECDC criteria HAI can be diagnosed on day 1 or day 2 if an invasive device was placed on day 1 or day 2 resulting in HAI before day 3.

**Table 2:** HAI definitions according to CDC/NHSN and ECDC criteria.

Type of HAI	CDC/NHSN^[^^19^^]^	ECDC^[^^20^^]^
ICU-acquired HAI	Event of the site-specific infection criterion occurs on or after the 3^rd^ calendar day of admission to an inpatient location where day of admission is calendar day 1	The onset of symptoms was on Day 3 or later (day of admission = Day 1) of the current admission ***or*** an invasive device was placed on day 1 or day 2 resulting in HAI before day 3
Pneumonia (PNU/PN)	Two or more serial chest imaging test with new and persistent infiltrate/consolidation/cavitation ***and*** signs and symptoms of pneumonia ***and*** positive clinical or microbiological diagnostic tests. PNU1- clinically defined pneumonia PNU2- pneumonia with specific laboratory findings PNU3- pneumonia in immunocompromised patients	Two or more chest X-rays or CT-scans with a suggestive image of pneumonia ***and*** signs and symptoms of pneumonia ***and*** positive clinical or microbiological diagnostic tests. PN1- Positive quantitative culture from minimally contaminated LRT specimen PN2- Positive quantitative culture from possibly contaminated LRT specimen PN3- Alternative microbiology methods PN4- Positive sputum culture or non-quantitative LRT specimen PN5- No positive microbiology (clinical criteria only)
Ventilator-associated pneumonia (VAP)/ Intubation-associated pneumonia (IAP)	Pneumonia where patient is on mechanical ventilation for >2 calendar days on the date of event, with day of ventilator placement being Day 1 ***and*** the ventilator was in place on the date of event or the day before.	Pneumonia where invasive respiratory device was present (even intermittently) in the 48 hours preceding infection.
Laboratory confirmed bloodstream infection (LCBI)/Bloodstream infection (BSI)	LCBI-1^¶^ recognized pathogen cultured from one or more blood cultures and organism cultured is not related to an another site LCBI-2 common skin contaminant cultured from 2 or more blood cultures + clinical symptoms and symptoms and positive laboratory results are not related to another site LCBI-3 patients ≤ 1 year of age	BSI*- One positive blood culture for a recognized pathogen -two positive blood cultures for a common skin contaminant and clinical symptoms Origin^¥^: C- Catheter related S- Secondary to another site U-Unknown
Central-line associated blood stream infection (CLABSI)/Catheter-related infections (CRI)	LCBI where central line (CL) or umbilical catheter (UC) was in place for >2 calendar days on the date of event, with day of device placement being Day 1 ***and*** the line was also in place on the date of event or the day before^†^	CRI1- local CVC related infection CRI2- general CVC related infection CRI3- microbiologically confirmed CVC-related BSI
Urinary tract infection (UTI)	SUTI- symptomatic UTI ABUTI^§^-Asymptomatic bacteremic UTI USI^‡^-Urinary System Infection	UTI-A- Microbiologically confirmed symptomatic UTI UTI-B- Not microbiologically confirmed symptomatic UTI UTI-C-Asymptomatic bacteriuria
Catheter-related UTI (CAUTI)/Device-associated UTI	An indwelling urinary catheter had been in place for > 2 days on the date of event (day of device placement is the day 1)	Urinary catheter was in place within the 48-hour period before onset of infection (even intermittently). The indwelling urinary catheter must have been in place within seven days before UTI was evident.

^*^Former HELICS BSI-A definition; BSI-B (single blood culture for skin contaminants in patients with central vascular catheter and adapted treatment) deleted since January 2009.

^¥^Primary BSI includes catheter-related BSI and BSI of unknown origin.

^¶^Primary LCBI includes CLABSI and non-CLABSI.

^§^Since 2009 ABUTI instead of ASB (asymptomatic bacteriuria).

^†^If a CL or UC was in place for >2 calendar days and then removed, the date of event of the LCBI must be the day of discontinuation or the next day to be a CLABSI.

^‡^Since 2015 USI-Urinary System Infection instead of OUTI (other urinary tract infections).

References. [19] CDC/HNSN definitions, 2014; [20] ECDC definitions, 2012.

The main difference in PN definitions is that CDC criteria contain pneumonia in immunocompromised patients (PNU3), which meet diagnostic criteria for PNU1 or PNU3 with only difference that is diagnosed in immunocompromised patients. PN in immunocompromised patients does not exist in ECDC criteria as separate category, and therefore can be categorized either as PN1-PN5 according to ECDC definitions. Difference between definitions was also observed for term mechanical ventilation. Pneumonia associated with mechanical ventilation is according to ECDC definitions named intubation-associated pneumonia (IAP) and it is diagnosed even if respiratory device is in place intermittently 48h preceding infection, unlike in CDC definitions according to which respiratory device needs to be constantly in place 48h before the onset of infection (inclusive of the weaning period).

According to both definitions, BSI is diagnosed when recognized pathogen is isolated from bloodstream or common skin contaminant cultured from 2 or more blood cultures. However, according to CDC definitions laboratory confirmed BSI (LCBI) must be a primary BSI, i.e. microorganism isolated from bloodstream must not be related with an infection at another site (secondary BSI). In contrast, ECDC’s BSI includes also secondary BSI as a possible source of BSI, together with “catheter” origin and “unknown” origin. Of note is that CDC’s primary BSI can be either CLABSI (Central line-associated BSI) or non-CLABSI (Non-central Line associated BSI), depending on the catheter use and that ECDC’s “catheter”and “unknown” origin of BSI comprise primary BSI which correspond to CDC LCBI CLABSI and non-CLABSI, respectively. Difference occurs also when it comes to BSI association with intravascular catheter. While according to CDC CLABSI is any LCBI where central line was in place for 48h before onset of symptoms, device-related BSIs according to ECDC include whole category of “catheter-related infections” which does not exist in CDC criteria. Catheter-related infections (CRI) comprise local catheter infection (CRI1), general catheter infection (CRI2) and microbiologically confirmed CVC-related BSI (CRI3). In practical terms, CDC’s CLABSI correspond to ECDC’s CRI3 (microbiologically confirmed CVC-related BSI), with the difference that ECDC’s CRI require microbiological confirmation of pathogen sameness between peripheral blood and central line which is not the case with CLABSI.

For urinary tract infections we observed two main differences. Firstly, ECDC definitions do not have category “urinary system infection” while CDC definitions do not have symptomatic not-microbiologically confirmed UTI (UTI-B). Secondly, CDC’s asymptomatic UTI (ABUTI) requires a positive blood stream specimen with at least one matching bacteria to the bacteria identified in urine sample, while ECDC’s diagnosis of asymptomatic bacteriuria (UTI-C) depends on presence of urinary catheter and number of positive urine cultures. The last implies that if indwelling urinary catheter was in place within 7 days before urine is cultured, one positive urine culture is enough to diagnose UTI-C, and if it in place within less than 7 days two positive urine cultures are necessary. Comparing device related UTI, intermittent use of urinary catheter according to ECDC and constant use according to CDC during 48h preceding symptoms of UTI is required to diagnose CAUTI according to EU and US definitions, respectively.

### HAI incidence

Of 406 patients, 107 (26.4%) acquired at least one HAI according to either criteria. A total of 107 infections (26%) were diagnosed according to CDC/NHSN surveillance definitions and 141 infections were diagnosed (35%) using ECDC criteria, and this difference was statistically significant (*X*^2^ = 6.711, *p* = 0.010). This represented an infection rate of 20.3/1000 patient-days for US and 26.8/1000 patient-days for European definitions, respectively.

### Definition concordance

Results of agreement analysis are summarized in Table 3. Comparison of overall HAI showed that US and European definitions differ in 34 discordant cases which were diagnosed according to ECDC but not according to CDC definitions resulting in almost perfect agreement between definitions (k = 0.90). When comparing case definitions for ICU-acquired HAI, 2 discordant cases were diagnosed by ECDC definitions but did not meet criteria for the timeframe of CDC ICU-acquired HAI since symptoms occurred within first two days of hospitalization during which patients had relevant invasive device in place. This resulted in almost perfect agreement with kappa coefficient 0.99.

**Table 3:** Agreement between HAI definitions.

Type of HAI	Criteria		Concordance (number of cases)				Kappa (95%CI)
	CDC/NHSN	ECDC	Either CDC/NHSN or ECDC	Both definitions	CDC/NHS BUT not ECDC	ECDC BUT not CDC/NHSN	
**Overall HAI**			141	109	0	34	0.90 (0.86–0.95)
**ICU-acquired HAI**	Event of the site-specific infection criterion occurs on or after the 3^rd^ calendar day of admission to an inpatient location where day of admission is calendar day 1	Infection occurred on day 3 after admission in the ICU ***or*** an invasive device was placed on day 1 or day 2 resulting in HAI before day 3	109	107	0	2	0.99 (0.81–1.00)
**Pneumonia**							
Overall	PNU1+PNU2+PNU3	PN1+PN2+PN3+PN4+PN5	51	51	0	0	1.00
Clinically defined	PNU1	PN4+PN5	4	3	1	0	0.86 (0.58–1.00)
	PNU1+PNU3	PN4+PN5	7	3	4	0	0.60 (0.25–0.95)
Microbiologically defined	PNU2	PN1+PN2+PN3	45	41	0	4	0.95 (0.90–0.99)
	PNU2+PNU3	PN1+PN2+PN3	48	47	0	1	0.99 (0.96–1.00)
Ventilator-associated pneumonia (VAP)	Continuous presence of device within 48h preceding pneumonia onset	Presence of device (even intermittently) within 48h preceding pneumonia onset	45	35	0	10	0.86 (0.78–0.94)
**Bloodstream infection (BSI)**							
Overall	Microorganism not related to infection at another site	Origin of BSI is”catheter”, “secondary to another site” or “unknown”	55	38	0	17	0.79 (0.70–0.89)
Primary BSI	Microorganism not related to infection at another site	Origin of BSI is”catheter” or “unknown”	38	38	0	0	1.00
	Microorganism not related to infection at another site	Origin of BSI is “unknown”	38	28	10*	0	0.63 (0.44–0.82)
CLABSI/CRI	Central line (CL) or umbilical catheter (UC) was in place for >2 calendar days on the date of BSI event	Clinical or microbiological evidence of relationship to central (CVC) or peripheral (PVC) vascular catheter	33	8	23^¶^	2^¥^	-0.01 (-0.20- (-0.18))
CLABSI/CRI3-CVC and CRI3-PVC	Central line (CL) or umbilical catheter (UC) was in place for >2 calendar days on the date of BSI event	Microbiologically confirmed CVC-related BSI	31	8	23	0	0.11 (0.0–0.22)
**Urinary tract infections (UTI)**							
Overall	SUTI+ABUTI+USI	UTI-A+UTI-B+UTI-C	35	18	0	17	0.66 (0.53–0.79)
Microbiologically confirmed symptomatic UTI	SUTI	UTI-A	17	17	0	0	1.00
CAUTI/Device-related UTI	The indwelling urinary catheter had been in place for > 2 days on the date of event (day of device placement is the day 1)	Urinary catheter was in place within the 48-hour period before onset of infection (even intermittently)	33	15	0	18	0.77 (0.34–1.0)

*All CRI infections (CRI1, CRI2 and CRI3).

^¶^CLABSI but not CRI since there was no clinical or microbiological confirmation of catheter origin of BSI.

^¥^Clinical evidence of catheter related BSI (CRI1-local CVC and PVC infection and CRI2-general CVC or PVC infection) but not CLABSI.

Considering pneumonia, we diagnosed 51 (12.6%) PN cases either by using US or European criteria, with the incidence rate of 9.7/1000 patient-days. We observed perfect concordance when comparing overall pneumonia cases (k = 1.00). However, discordance occurred when PN divided into subcategories of clinically defined PN and microbiologically confirmed PN. Higher agreement was observed for microbiologically confirmed PN (k = 0.99) than for the clinically defined PN (k = 0.86). Moreover, in our study there were 6 immunocompromised patients out of whom 3 developed PN and all of them were microbiologically confirmed. Since PN in immunocompromised patients does not exist in ECDC criteria these cases had to be qualified either as microbiologically confirmed or clinically defined PN. When PNU3 were qualified into PNU2, agreement was almost perfect (k = 0.99) but when qualified into PNU 1 (clinically defined) agreement was only substantial (k = 0.60).

Regarding the pneumonia associated with mechanical ventilation, 45 VAP cases were diagnosed either by US or European criteria with incidence 19.1/1000 ventilator-days. Ten discordant cases were identified, defined according to ECDC but did not meet CDC criteria, resulting in near perfect agreement (k = 0.86).

Fifty five BSI cases were diagnosed by using either criteria, 38 when using CDC (IR = 7.23/1000 patient-days) and 55 when using ECDC definitions (IR = 10.5/1000 patient-days). When considering overall BSI cases agreement was only substantial (k = 0.79) due to 17 discordant cases. All these 17 cases were secondary BSI, therefore when we compared only primary BSIs [n = 17 (30.9% of all BSI cases)] agreement was perfect. However, when BSI related to central/peripheral vascular catheter reported separately as CRI (as recommended by the last ECDC protocol update) [n = 11 (20% of all BSI cases)], agreement for BSI was only moderate (k = 0.60).

Considering device-related BSI, 31 case out of 38 primary BSI were CLABSI according to CDC definitions since CVC was in place 48h preceding infection (81.6%; IR = 7.3/1000 CVC-days), while 10 cases out of 38 primary BSI were ascertained as CRI by applying ECDC criteria for microbiological or clinical association of positive blood culture with CVC (26.3%; IR = 2.3/1000 CVC-days). However, only 8 cases met the criteria of both definitions, leaving 23 discordant cases, of which 2 were local and generalized catheter-related infections (CRI1 and CRI2) with no positive blood culture and therefore not CLABSI. Due to small number of CRI cases and major discrepancies between definitions, no agreement better than chance was observed between them (k = -0.01). Slight agreement was observed when compared CLABSI with microbiologically confirmed CVC-BSI (CRI3) (k = 0.11).

Thirty five episodes of UTI were observed according to ECDC definitions and 18 UTI according to CDC definitions during 4860 days spent with urinary catheter (IR = 7.2/1000 and 3.7/1000 device days, respectively). Eighteen cases of asymptomatic UTI (UTI-C) were diagnosed following ECDC criteria, of which 17 were not considered ABUTI according to CDC definitions since there was no positive blood culture with at least one matching bacterium isolated necessary to diagnose ABUTI, resulting in only one concordant case of asymptomatic UTI and moderate agreement between UTI definitions (k = 0.66). However, when comparing microbiologically confirmed symptomatic UTI there were 17 concordant cases and no discordant cases and perfect agreement (k = 1). Considering catheter-related UTI (CAUTI), 15 of 18 (83.3%) urinary infections were CAUTI according to CDC definitions and 33 out of 35 ECDC’s UTI cases (94.3%) were catheter-associated UTIs. Due to 17 discordant asymptomatic UTI cases, and 1 ECDC’s UTI-A case which had catheter in place intermittently for 48h preceding infection, agreement between definitions was also moderate (k = 0.77).

## Discussion

In order to achieve effective HAI control globally, adoption and adherence to standardized surveillance methods and case definitions by national and regional surveillance networks are essential since they allow reliable interpretation and comparison of HAI estimates. This is particularly important for the eastern European enlargement countries whose national PSS results will be reported within the second EU-wide PPS, and which, for this purpose, transferred from CDC to ECDC definitions. Since the infection control in these countries face many financial and methodological issues [22], the impact of this transition in such a country should be addressed. We therefore performed a study to evaluate the impact of change of HAI definitions on HAI estimates in the sample of ICU trauma patients.

Main differences observed between US and ECDC definitions comprised difference for HAI case definition, difference in definition criteria for several HAI localizations (PN, BSI and UTI) and difference in definition for device-associated infections (VAP, CLABSI, CRI and CAUTI). First refers to the device use during the first two days since according to EU definitions ICU acquired infection can occur on Day 1 or Day 2 if invasive device was in place on Day 1 or Day 2 [20]. However, when these definitions applied to ICU patients who are exposed to numerous invasive devices from the day one, few discordant cases occurred, implying that this change may be even less apparent in the non-ICU wards.

Change in definitions for specific infection localizations were observed for PN, BSI and UTI. For PN, overall concordance of the definitions was perfect with no discordant cases until they were divided into clinically defined and microbiologically confirmed PN. More cases of clinically defined PN were diagnosed according to CDC, while more cases of microbiologically confirmed PN were diagnosed using ECDC definitions. This may have occurred because we classified ECDC’s PN2 (“quantitative culture from possibly contaminated low respiratory tract specimen” such as endotracheal aspirate) as microbiologically confirmed PN, as recommended by ECDC protocol [20]. Moreover, agreement between definitions remained much higher for MO confirmed PN than for clinically defined PN even when PNU3 cases (“PN in immunocompromised patients”) classified into MO defined. Similar results were shown by *Hansen S*. *et al* in concordance study conducted in seven European countries as a part of HELICS/IPSE transition from CDC to ECDC [23]. All this can imply that microbiological confirmation is very important when diagnosing PN since specific and more objective criteria result in better agreement between definitions and are easier to apply in more standard manner, regardless of patient’s immune status.

In our study, agreement between definitions for BSI depended mostly on BSI category classification i.e., whether secondary BSI and catheter related BSI are reported separately by ECDC definitions or as overall BSI. Despite of not reporting secondary BSI within PPS due to focusing on prevention of primary BSI, CDC has recently established a guidance on classification of secondary BSI for the purpose of distinction and assurance of primary BSI and avoidance of possible misclassification of BSI types [24]. When considering only primary BSI (LCBI in CDC definitions and BSI of “unknown” or “catheter” origin in ECDC criteria) agreement was perfect since both definitions comprised only BSI cases not related to an infection at another site. Moreover, slightly higher overall incidence rate observed when using ECDC definitions can be attributed to reporting of secondary BSI according to ECDC definitions. The similar can be expected when obtaining results from national PPS study, since secondary BSI accounts for almost third (28.8%) of all BSI in European acute care hospitals [25]. For eastern European counties which still have mandatory reporting of HAI within the list of mandatory reportable diseases, this will also inflate the number of reported BSI cases and has to be taken into consideration when interpreting the national surveillance data.

Since 2009 American definitions for UTI were subject to numerous changes including removal of asymptomatic bacteriuria (ASB) and inclusion of asymptomatic bacteremic UTI [26]. Exclusion of broad ASB definition which relied mostly on duration of usage of urinary catheter, and inclusion of more objective ABUTI definition which requires a positive blood specimen with at least one bacteria same to the one isolated from urine culture, along with increment of threshold for detecting uropathogens to ≥ 10^5^ when diagnosing SUTI, greatly increased specificity of UTI diagnosis. However it resulted in limited longitudinal [27] and inter-network comparison of UTI and CAUTI [23].

Difference for device-related HAI was most apparent when diagnosing BSI related to vascular device. While CLABSI is a term used by DCD/NHSN, defined as a primary infection developed in patient with a CVC in place for more than 48h before the onset of BSI that is not related to infection at another site and does not require catheter tip culture or peripheral blood culture as a criterion [19], CRI-BSI requires specific laboratory tests such as catheter tip culture and different time to positivity as a condition to be diagnosed [20]. Consequently, incidence of BSI associated with CVC use can be rather over- or underestimated, which can have major impact on comparison of prevalence estimates of BSIs associated with use of intravascular devices and its prevention strategies [28]. Higher concordance was observed between VAP definitions (k = 0.86) since 10 pneumonia cases were diagnosed according ECDC definitions which could not be included according US criteria since they do not accept intermittent use of MV in relation with pneumonia (excluding weaning period). This is higher comparing to EU concordance study where they observed fewer discordant cases and can be explained by different weaning practices and higher extubation failure rate followed by reintubation in trauma patients [29].

The practical significance of the results of this study refers to the better understanding of the difference between the US and EU HAI definitions, not only at local but at the national level too as our national surveillance system has some specific characteristics. Briefly, 25 regional public health institutes collect the HAI data from hospitals under their authority and after logical control of the data, they forward them to the National Institute of Public Health which publish the national HAI data with an inter-facility comparison for the year when the surveillance was conducted and for the five previous years. Since from January 2018 ECDC HAI definitions are in use for the purpose of mandatory notification and surveillance of HAI, the HAI incidence is expected be to be higher compared to previous year. It is important to recognize that this will not be a reflection of a worse epidemiological situation as it is rather an effect of the differences between US and European definitions, especially for secondary BSI, urinary tract infections and CLABSI.

Limitation of the study is that both sets of definitions were subject to additional changes after study was conducted. Important additional change is that according to ECDC 5.3 protocol update [16] one definite chest x-ray or CT scan for the current episode of PN is sufficient for patients with underlying cardiac or pulmonary disease if comparison with previous x-rays is possible while according to CDC definitions and 4.3 ECDC protocol two or more serial x-rays are required for these patients [20]. This is not likely to affect results when ICU patients are considered since they all have several control chest x-rays as well as first x ray from the admission to ED. Also, the design of target-oriented prospective surveillance, i.e., choosing patients in ICU due to significant exposure to risk factors, may not reflect actual changes which would have occurred at other medical ward. However, population of ICU patients give rise to important strengths of the study which are that ICU setting allows constant monitoring and active search for the infection signs and symptoms and allowed us measuring hours from admission when diagnosis HAI rather than days, which all facilitated accurate diagnosis of HAI.

## Conclusions

In conclusion, excellent concordance between CDC/NHSN and ECDC definitions was observed for primary BSI and laboratory confirmed PN and microbiologically-confirmed symptomatic UTI. Major differences in definitions were found for clinically defined PN, overall BSI, and CLABSI. Practical implications are that microbiological confirmation of pneumonia and urinary infection should be facilitated since it is associated with better concordance of diagnosis and that comparison of BSI should be done with emphasis on whether secondary BSI and catheter related BSI are reported separately or as overall BSI.

## Supporting information

S1 File Minimal dataset underlying research findings. (XLS)
